# NKT Cell Networks in the Regulation of Tumor Immunity

**DOI:** 10.3389/fimmu.2014.00543

**Published:** 2014-10-28

**Authors:** Faith C. Robertson, Jay A. Berzofsky, Masaki Terabe

**Affiliations:** ^1^Vaccine Branch, Center for Cancer Research, National Cancer Institute, National Institutes of Health, Bethesda, MD, USA

**Keywords:** NKT cell, tumor immunology, immune regulation, immune network, NKT cell subsets

## Abstract

CD1d-restricted natural killer T (NKT) cells lie at the interface between the innate and adaptive immune systems and are important mediators of immune responses and tumor immunosurveillance. These NKT cells uniquely recognize lipid antigens, and their rapid yet specific reactions influence both innate and adaptive immunity. In tumor immunity, two NKT subsets (type I and type II) have contrasting roles in which they not only cross-regulate one another, but also impact innate immune cell populations, including natural killer, dendritic, and myeloid lineage cells, as well as adaptive populations, especially CD8^+^ and CD4^+^ T cells. The extent to which NKT cells promote or suppress surrounding cells affects the host’s ability to prevent neoplasia and is consequently of great interest for therapeutic development. Data have shown the potential for therapeutic use of NKT cell agonists and synergy with immune response modifiers in both pre-clinical studies and preliminary clinical studies. However, there is room to improve treatment efficacy by further elucidating the biological mechanisms underlying NKT cell networks. Here, we discuss the progress made in understanding NKT cell networks, their consequent role in the regulation of tumor immunity, and the potential to exploit that knowledge in a clinical setting.

## Introduction

Effective tumor immunosurveillance is mediated by players from both the innate and adaptive immune systems. Innate immune cells (including macrophages, neutrophils, dendritic cells (DCs), and natural killer cells) rapidly respond to an immunological threat such as cancer. However, it is important that information from this front-line defense be transmitted to the adaptive immune system. Adaptive immune cells, including T and B lymphocytes, mediate tumor immunity in a more precise and lasting manner via antigen-specific and effector memory responses. A few key players in immunosurveillance lie at the interface of innate and adaptive immunity and facilitate this transition, one of which is natural killer T (NKT) cells. In an innate fashion, NKT cells have the ability to react quickly, producing a broad range of cytokines within minutes to hours of antigenic stimulation and also activate antigen-presenting DCs. This rapid effect can trigger powerful innate and acquired immune responses both directly and indirectly. Thereby, NKT cells bridge two major defense systems to modulate immunity and are important in influencing host immune responses to cancer and other diseases. Here, we review the current understanding of NKT cells regarding their role in enhancing or suppressing tumor immunity, as well as ongoing strategies using NKT cells for cancer immunotherapy and discuss future approaches that might optimize NKT cell-based anti-tumor responses.

## Natural Killer T Cells

Natural Killer T cells are a heterogeneous lymphoid population that exhibits characteristics from both the innate and adaptive arms of the immune system. Similar to innately functioning NK cells, these lymphocytes react quickly to stimuli and produce an array of cytokines and chemokines to modulate the immune response (1, 2). NKT cells respond in an antigen-specific manner through a true T cell receptor (TCR). However, unlike TCRs from conventional T cells that recognize one epitope (or a few at most), a single TCR from NKT cells can react with numerous antigens, both self and foreign. Thus, even though the absolute frequency of NKT cells is low (e.g., ~1% in mouse spleen), their number is high in the context of T cells specific for one antigen, high enough to initiate a significant immune response (3–5). Additionally, they differ from conventional T cells that recognize peptide antigen presented by class I or II major histocompatibility complex molecules (MHCI or II) on antigen-presenting cells (APCs). Instead, NKT cells interact with lipid antigens presented by an MHC-like molecule known as CD1d. In fact, because many NKT cells do not express NK cell markers, the defining characteristic of NKT cells is now CD1d restriction, and NK-like markers are primarily used to characterize subsets (6).

Natural killer T cells may have both immune enhancing and immunosuppressive roles. Upon antigenic stimulation, NKT cells are able to produce a range of cytokines including interferon-gamma (IFN-γ), interleukins (IL)-2, -4, -10, -13, -17, -21, and 22, granulocyte-macrophage colony-stimulating factor (GM-CSF), and tumor necrosis factor-alpha (TNF-α) (7–9). The cytokine profile produced differs slightly among subsets, but interestingly, NKT cells can simultaneously secrete Th1/pro-inflammatory (e.g., IFN-γ, TNF-α) and Th2/anti-inflammatory (IL-4, IL-10, IL-13) cytokines. The cytokines secreted activate other immune cells such as NK cells, T cells, and B cells, potentiating a cascade of cytokine and chemokine production and galvanizing additional cytolytic populations to mediate tumor surveillance (2). Because, NKT cell activation contributes to DC maturation (10), and in turn enhances both priming and boosting of CD8^+^ T cells, co-administering peptide, or protein antigen with NKT cell agonists has an adjuvant effect. This activity can be channeled toward improving vaccines for cancer, infectious diseases, and other therapeutic settings (10–12).

**Figure 1:**
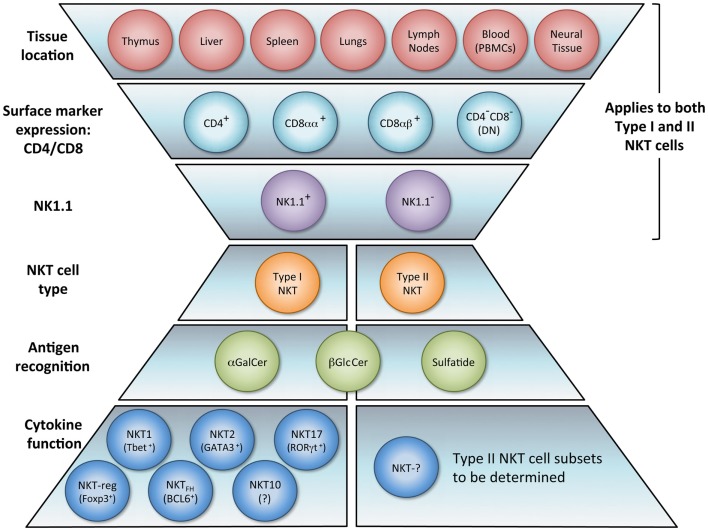
**NKT cell subsets**. The natural killer T (NKT) cell population encompasses several phenotypically and functionally different subpopulations. Tissue location and surface markers (CD4, CD8, and NK1.1) are defining characteristics that broadly divide NKT cells and contribute to functionality. Differences in TCR rearrangements allow separation into two major subsets, type I and type II. Type I NKT cells express a semi-invariant TCRα chain, while type II NKT cells display a more diverse repertoire. It has been proposed that these NKT cell subsets recognize distinct lipid antigens. The prototypic antigen able to activate all type I NKT cells is α-GalCer. Type II NKT cells recognize a greater variety of antigens, one being sulfatide. Though these two subsets have been reported to recognize some common antigens, e.g., β-GlcCer, the biochemical structure is slightly different between the antigen recognized by type I versus type II NKT cells. Lastly, type I NKT cells are functionally heterogeneous. NKT1, NKT2, NKT17, NKTreg, NKT_FH_, and NKT10 subsets have been described. Overall, an *in vivo* NKT cell response likely depends on which subsets are activated.

The NKT cell population is composed of several phenotypically and functionally different subpopulations, and can be characterized further according to tissue location, surface markers, specific TCR usage, and effector functions (Figure 1). Surface markers CD4 and CD8 broadly divide NKT cells, where a majority is CD4^+^ (approximately 90% in mice) and the remainder is CD4^−^CD8^−^ double negative (DN). In human beings, additional minute populations of CD8αα and CD8αβ NKT cells exist (13). Activated human DN NKT cells predominantly produce cytokines that instigate a Th1 response, while CD4^+^ NKT cells generate both Th1 and Th2 cytokines (1, 14, 15). NKT cells can also be subdivided into NK1.1^+^ or negative, with different functional consequences (16). Aside from CD4 and CD8 expression, functional differences in the nature and magnitude of the NKT cell response can arise from developmental differentiation and tissue location. For instance, NKT cells from human peripheral blood generate cytokines similar to those isolated from spleens but differ significantly from NKT cells in the thymus or cord blood (17). Peripheral blood NKT cells are also distinct from liver NKT cells in terms of surface marker expression (18). Because, human NKT cells from peripheral blood are commonly used as a surrogate for systemic NKT cell analysis, this discrepancy suggests that current protocols analyzing NKT cell number and functionality may be misleading. NKT cell frequency and expression of cell surface proteins has been shown to differ between various sites in the small and large intestine as well (19). Functional differences of NKT cells from different tissues have also been demonstrated in the context of tumor (20). These comparative analyses provide insight that NKT cell tissue location should be considered when extrapolating information from experimental or clinical data, and underscores that a clearer description of tissue-specific characteristics of NKT cells is needed.

Heterogeneity of TCR rearrangements has allowed NKT cells to be separated into two categories, type I and type II (as described below). In the context of tumor immunity, these subsets have been shown to differentially impact innate and adaptive immune cell populations. Type I NKT cells are usually associated with the promotion of tumor immunity whereas type II NKT cells seem to suppress it (21–27).

### Type I NKT cells

Type I NKT cells express a semi-invariant TCRα chain (Vα14-Jα18 TCR in mice, Vα24-Jα18 in human beings) paired with a limited repertoire of Vβ chains (primarily Vβ8, 7 and 2 in mice, Vβ11 in human beings) and are consequently referred to as invariant or iNKT cells. In type I NKT cells, it appears that a combination of activation variables dictates NKT cell function: the affinity of the antigen presented to the NKT TCR; the presence of costimulatory molecules; and the tissue environment in which the interaction takes place (7, 28). The prototypic antigen for type I NKT cells is α-galactosylceramide (α-GalCer or KRN7000), a synthetic form of a glycolipid isolated from a marine sponge (29, 30). Type I NKT cells also recognize microbial glycolipids and self-antigens, e.g., *Sphingomonas* and *Ehrlichia* lipids, lyso-phosphatidylcholine (lyso-PC), and isoglobotrihexosylceramide (iGb3) (31–35). α-GalCer is a potent activator of all type I NKT cells, causing them to produce copious amounts of IFN-γ, which helps activate both CD8^+^ T cells and APCs (36). NKT cells specifically stimulate DCs through the CD1d-TCR complex and CD40–CD40L interaction, which induces DC maturation and IL-12 secretion (37, 38). IL-12 stimulates both NK, NKT, and other T cells to produce more IFN-γ, and the two cytokines together significantly impact the activation of downstream effector populations such as NK cells, CD8^+^ T cells, and γδ T cells (39). NKT cell activation also causes DCs to upregulate costimulatory receptors (e.g., CD70, CD80, and CD86). CD70 expression by DCs is essential for cross-priming CD8^+^ T cells to promote adaptive immunity (40–42). IL-2 produced by activated NKT cells induces the proliferation of memory CD4^+^ T helper 1 (Th1) and Th2 cells (43). Additionally, because differentiation of CD4^+^ T cells into T helper cell subsets depends on the cytokine milieu, cytokines from NKT cells may facilitate polarization into Th1, Th2, and/or Th17 subsets. Having these innate and acquired immune reactions occur simultaneously is integral for a potent immunological response, especially for eradication of tumor masses, which frequently contain both MHC-negative cells (targeted by NK cells) and MHC-positive cells (targeted by CD8^+^ T cells) (44).

Of recent interest are unique cytokine producing subsets of type I NKT cells, particularly those making IL-17. A study analyzing subsets according to tissue origin and CD4 and NK1.1 marker expression found significant diversity of cytokine production by distinct subsets, especially CD4^−^NK1.1^−^ NKT cells that produce high levels of IL-17 (16, 45). IL-17 has potent pro-inflammatory functions including the induction of IL-6 and TNF-α, as well as the recruitment and enhancement of neutrophils. Analogous to CD4^+^ Th17, primary producers of IL-17, this NKT cell lineage constitutively expresses the RORγ-t transcription factor, as well as IL-23R (46). However, the NKT17 population was isolated from naïve animals without priming, and was able to secrete IL-17 as soon as 2–3 h following antigen stimulation, whereas naïve CD4^+^ T cells must undergo a differentiation period of a few days before antigen can polarize the cell into Th17 phenotype and elicit such a response. Other reports have further described this NKT cell subset by IL-17R expression and lack of NK1.1 expression, or added that mature differentiation of CD44^+^CD4^−^NK1.1^−^cells manifests in stage 2 of thymic development (46, 47). Additional type I NKT cells have been characterized by their transcription factor expression and effector functions in a manner comparable to other CD4^+^ T helper subsets [Th1, Th2, Tregs, T follicular helper cells (T_FH_)] (48–50). Lee et al. report that NKT1, NKT2, and NKT17 cells are programed intrathymically to elicit a particular cytokine profile (49). Others showed that Foxp3^+^ type I NKT cells behave similarly to Tregs and suppress conventional CD4^+^ T cell proliferation in a contact-dependent and antigen-independent manner (48). A subset of IL-10-producing NKT cells was recently reported by Sag et al. (51). This demonstrates diverse lineages of molecularly distinct type I NKT cell subsets that differ functionally in their production of particular cytokines. Our incomplete knowledge of the heterogeneity within type I NKT cells has likely hindered progress in harnessing the true potential of NKT cells; NKT cell responses *in vivo* likely depend on which subsets are activated. A better understanding of NKT cell subcategories could inform more selective and focused mechanisms for immunological intervention.

### Type II NKT cells

In contrast, CD1d-restricted NKT cells not expressing the semi-invariant TCR are known as type II. This subset recognizes glycolipid antigens distinct from those recognized by type I NKT cells and is less well characterized. For those reactive to sulfatide, a myelin-derived glycolipid found abundantly in the nervous system, the type II NKT TCR repertoire shows preferential use of alpha gene segments from Vα1 and Vα3, paired with Vβ8.1/Vβ8.3 (52). Because, type II NKT cells do not utilize the Vα14-Jα18 gene segment, they can be studied by comparing wild type (WT) mice that have both type I and II NKT cells, to Jα18 knockout (KO) mice that lack type I NKT cells but retain type II, and to CD1d KO mice, which are deficient in all NKT cells. Type II NKT cells have also been studied with TCR transgenic mice expressing the 24αβ-TCR from the CD4^+^ type II NKT cell hybridoma VIII24 that expresses a Vα3.2 and Vβ9 rearrangement and is not reactive to sulfatide (53). Additionally, because NKT cells constitutively express IL-4 mRNA transcripts, the use of IL-4 GFP enhanced transcript (4get) mice has introduced another type II NKT subset (54, 55). Liver NKT cells from 4get Jα18-deficient mice were sorted to obtain TCRβ^+^GFP^+^ α-GalCer/CD1d tetramer-negative cells. This population produced IFN-γ when CD1d-expressing bone marrow-derived DCs presented several types of lipids, including β-d-glucopyranosyl ceramide (β-GlcCer), β-GalCer, and Lyso-PE. The pool of β-GlcCer reactive type II NKT cells did not respond to sulfatide and favored TCR gene segments from Vα8 and Vβ8.1/8.2 (55). It is apparent that there are many distinct populations within the type II NKT cell division; however, even with the recent methodological advances that have enhanced our ability to study this non-invariant subset, much less is known about type II than type I NKT cells.

## NKT Cell Enhancement of Tumor Immunity

**Figure 2:**
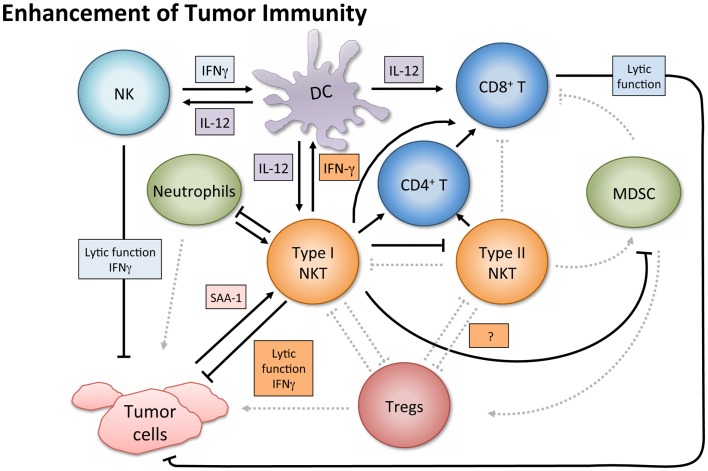
**Enhancement of tumor immunity**. Upon antigenic stimulation, type I NKT cells produce copious amounts of IFN-γ, which helps activate both CD8^+^ T cells and DCs. NKT cells specifically induce DC maturation by engaging the CD1d-TCR complex and CD40–CD40L interaction. DCs then upregulate costimulatory receptors essential for the cross-priming of CD8^+^ T cells to promote adaptive immunity. Additionally, IL-12 production by DCs stimulates NK, NKT, and other T cells to produce more IFN-γ and the two cytokines together significantly impact the activation of downstream effector populations. Both type I and type II NKT cells have been shown to enhance proliferation of memory CD4^+^ T cells, which can help CD8^+^ T cells as well. NK and type I NKT cells are able to directly lyse tumor cells utilizing various mechanisms which include perforin, granzyme, and FasL. Type I NKT cell stimulation can also enhance tumor immunity by hindering immunosuppressive populations. Type I NKT cells regulate effects of type II and expansion of MDSCs. Tumor-induced inflammatory proteins like serum amyloid A 1 (SAA-1) have been shown to increase neutrophilic CD1d-dependent stimulation of type I NKT cells, which then mitigates the detrimental activity of suppressive neutrophils by hindering production of IL-10 and enhancing IL-12.

In tumor immunosurveillance, NKT cells can directly kill malignant cells, or combat cancer indirectly via activation of additional immune cells (Figure 2). Type I NKT cells employ various mechanisms of cytolytic activity. For instance, both mouse and human NKT cells can directly lyse tumor cells by a perforin-dependent mechanism (56), and intracellular granzyme B expression may also potentiate cell killing (57). Tumor cells expressing CD1d may be especially susceptible to direct NKT cell lysis, as shown *in vitro* (58–60). This pattern is consistent with *in vivo* observations (61, 62) and there is evidence that high CD1d expression levels correlate with lower metastasis rates (60).

While type I NKT cells are undoubtedly capable of mediating tumor lysis directly, several studies in both human beings and mice demonstrate NKT cell-mediated anti-tumor effects that are independent of lytic capacity and largely dependent on activating other innate and adaptive immune cells (20, 51, 63, 64). This recruitment of anti-tumor cytolytic cell populations primarily involves initiating Th1 cytokine cascades. As mentioned above, α-GalCer was the first NKT cell ligand identified and is a potent activator of type I NKT cells. It showed clinical potential when application of its synthetic form (KRN7000) increased survival in mice bearing the B16 melanoma (29, 58). The copious amount of IFN-γ released upon α-GalCer stimulation appears necessary for tumor protection (12, 65). In lung and liver metastasis models, production of IFN-γ by NKT cells and NK cells was absolutely required to obtain anti-metastatic activity of α-GalCer, and IL-12 and IL-18 were necessary to achieve optimal serum IFN-γ induction and tumor immunity (63). Type I NKT cells can also mediate tumor immunosurveillance in the absence of exogenous antigen. In a model of MCA-induced fibrosarcoma, Jα18 KO mice lacking type I NKT cells had a greater susceptibility to the disease. Adoptive transfer of liver DN type I NKT cells from WT into Jα18 KO mice restored the NKT cell population and rescued tumor immunity. However, CD1d KO mice were not protected upon NKT cell transfer, demonstrating that the protection was CD1d-dependent (20, 22). Additionally, only the DN liver subset conferred protection in this model; the CD4^+^ subset of liver NKT cells were not protective, and when type I NKT cells from the thymus were adoptively transferred, only slight protection was observed. Notably, the thymic NKT cells produced more IL-4 than those from liver or spleen. Thus, even in the absence of exogenous antigenic stimulation, it is becoming clearer that different subsets of NKT cells play distinct roles in immunosurveillance (66).

While surface marker expression and anatomical origin may influence NKT cell function, different antigens can also impact the immunological capacity of NKT cells. Examining both the cytokine profile and tumor protection capacity within a panel of type I NKT agonists, there was a strong correlation between the Th1 profile (in terms of IFN-γ:IL-4 ratio or IFN-γ production) and magnitude of protection in the mice from tumor growth in CT26 lung metastasis model (67). Least effective in the panel was OCH, which induced some IFN-γ but was the most Th2 skewed (IL-4 and IL-13) cytokine response in this set. OCH still suppressed tumor growth, but less than antigens eliciting greater Th1 skewing. Because, IFN-γ production appears to be the best correlate for tumor protection, and because, simultaneous NKT cell production of both Th1 and Th2 type cytokines may lessen the beneficial Th1 profile effects, multiple groups have tried enhancing α-GalCer to heighten IFN-γ production (68–72). A synthetic C-glycoside analog, α-C-galactosylceramide (α-C-GalCer), stimulated prolonged production of IFN-γ and IL-12 *in vivo*, and decreased production of the Th2 cytokine IL-4 compared with α-GalCer (69), as confirmed in our hands as well (O’Konek unpublished). This enhanced protection against B16 melanoma pulmonary metastases (69). An induced fit antigen with enhanced binding to CD1d, naphthylurea (NU)-α-GalCer, heightened Th1 biased cytokine skewing, and conferred better tumor protection than α-GalCer (72). In the same study, a type I NKT cell agonist that stimulated a weak Th2 biased cytokine production (xylo-α-GalCer) had no protective effect. Analog 7DW8-5, which possesses a fluorinated benzene ring, improved NKT cell stimulation, and exhibited an adjuvant effect superior to that of α-GalCer on HIV and malaria vaccines (73). Analogs containing phenyl group(s) on the lipid tail of α-GalCer also seem to steer NKT cells toward a Th1-skewed response (70), and exhibited more potent anticancer activities than α-GalCer in mice by inducing neither NKT cell anergy nor expansion of myeloid-derived suppressor cells (MDSCs) (70, 74). Overall, superior Th1-biasing antigens have shown enhanced tumor protection and may warrant translation to the clinic (72).

Aside from augmenting protective cell responses in both arms of the immune system, NKT cells can enhance tumor immunity by altering the effects of immunosuppressive cells. For instance, in a study on influenza A virus infection, the absence of type I NKT cells resulted in the expansion of MDSCs, which suppressed CD8^+^ T cell immune responses (75). Adoptively transferred liver NKT cells restored the WT phenotype, and the result was shown to be CD1d-dependent. Although the protection did not require stimulation of transferred type I NKT cells by an exogenous antigen, activating NKT cells with α-GalCer reversed MDSC-mediated suppression in a CD40–CD40L manner. Because some MDSCs (e.g., those activated by tumor-derived IL-1β) impair NK cell development and function (76), having NKT cells present to prevent MDSC expansion may enhance NK cell activity as well. NKT cells can also counter tumor-driven immunosuppressive neutrophils (77). Tumor-induced inflammation causing secretion of serum amyloid A1 (SAA-1) induced differentiation of suppressive IL-10-producing neutrophils. However, SAA-1 also increased interaction between neutrophils and type I NKT cells. The suppressive neutrophils expressed both CD40 and CD1d and the presence of SAA-1 triggered a neutrophilic CD1d-dependent activation of type I NKT cells comparable to stimulation with α-GalCer-pulsed neutrophils. Type I NKT cells not only mitigated the detrimental effect of neutrophils by hindering production of IL-10, and enhancing IL-12, but also restored proliferation of antigen-specific CD8^+^ T cells. Because this effect was proportional to the numbers of type I NKT cells added *in vitro*, it would be interesting to see if greater quantities of NKT cells *in vivo* would shift the equilibrium in immune reaction to SAA-1 further, decreasing IL-10 mediated suppression, and restoring tumor immunity. This balance and crosstalk underscores the importance of type I NKT cells in the regulation of tumor immunity and introduces an opportunity to exploit NKT agonists in tumor settings where inflammatory proteins like SAA-1 are released.

## NKT Cells in Suppression of Tumor Immunity

**Figure 3:**
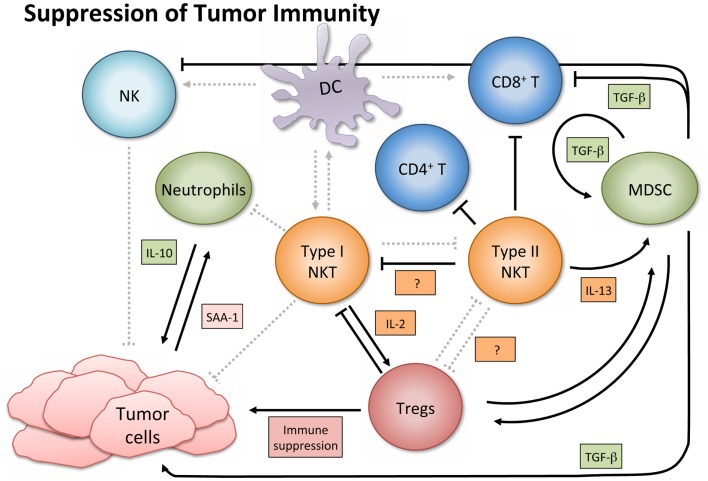
**Suppression of tumor immunity**. Activated type I NKT cells have been shown to support immunosuppressive Tregs through IL-2 production, and are then suppressed by Tregs in a cell-contact-dependent manner. Treg cells can then suppress CD8^+^ and CD4^+^ T cells and NK cells (not shown). Sulfatide-reactive type II NKT cells suppress CD8^+^ T cells and inhibit proliferation of naïve, but not memory, CD4^+^ T cells. Type II NKT cells also suppress Type I, and while the exact mechanism in cancer settings remains unknown, the mechanism in a con-A-induced hepatitis model is believed to involve pDCs. Type II NKT cell production of IL-13 functions with TNF-α from other cells to increase production of TGF-β by a CD11b^+^Gr1^+^ population known as myeloid-derived suppressor cells (MDSCs). MDSCs not only directly support tumor growth with TGF-β production but also suppress other immune cells (e.g., CD8^+^ T and NK cells), feed into an autocrine loop to enhance development of additional tumor-associated MDSCs, and aid in expansion of Tregs. When tumor-induced inflammatory proteins like SAA-1 stimulate suppressive IL-10 producing neutrophils, and type I NKT cells are not activated to alter that reaction, increased levels of IL-10 can induce Treg cells and potentiate further tumor growth.

In contrast to their immune enhancing role in cancer, NKT cells, especially type II, have displayed immunosuppressive activity in tumor immunology (Figure 3). A series of studies using multiple tumor models demonstrated that type II NKT cells were sufficient for down-regulation of tumor immunosurveillance, within the context of type I and type II NKT cells (26, 27, 61, 78). CD4^+^ type II NKT cells produced more of IL-13 and IL-4 than type I cells, and the NKT cell-dependent IL-13 was necessary for tumor recurrence in a growth-regression-recurrence pattern 15-12RM fibrosarcoma tumor model (24). The immunosuppressive effect seemed mediated by the sulfatide-reactive subset of type II NKT cells, as sulfatide treatment enhanced tumor growth in a CD1d-dependent manner (27). IL-13 initiated signaling through the IL-4R-STAT6 pathway, which together with TNF-α led to an increase in production of transforming growth factor beta (TGF-β) by a CD11b^+^Gr1^+^ population, sometimes called MDSCs (25, 79). TGF-β is a pleiotropic cytokine with three isoforms and TGF-β1 has been shown to down-regulate cytotoxic T cell lymphocyte (CTL) activity and in turn repress tumor immunosurveillance in several mouse tumor models. Blocking TGF-β or depleting Gr-1^+^ cells *in vivo* prevented tumor recurrence (25). Type II NKT cells were also shown to suppress immunity against CD1d-transfected B cell lymphoma; amounts of IL-13, TGF-β, and MDSCs correlated with immune suppression (61).

Myeloid-derived suppressor cells accumulate in the bone marrow and lymphoid organs of cancer patients and can counteract T cell responses. Because type II NKT cells appear to enhance MDSC activity, manipulating the NKT-MDSC immune axis has therapeutic implications. However, what works in one tumor model does not always translate to others. We investigated the anti-tumor effects of neutralizing TGF-β in conjunction with a peptide vaccine (80). In a pre-clinical study using a TC1 tumor in C57BL/6 mice, a pan-inhibitor of all three TGF-β isoforms had no impact as a single agent, suggesting that the mechanism of immunosuppression was different from that in 15-12RM. However, TGF-β neutralization significantly and synergistically improved cancer vaccine efficacy by reducing growth of primary tumors, and increasing tumor antigen-specific CTLs (80). Similar synergy between a vaccine and a TGF-β antagonist has been reported in multiple tumor models with multiple vaccine platforms (81–83). Currently, because the third isoform (TGF-β3) is hypothesized to have beneficial physiological effects in cancer (84), we are investigating the role of different TGF-β isoforms in the tumor setting and in conjunction with a cancer vaccine that contains a tumor antigen and a type I NKT agonist. It may not be necessary to block all three isoforms of TGF-β to promote tumor immunity or enhance cancer vaccine efficacy (Terabe et al., manuscript in preparation). This could be utilized in a push-pull approach (85) to maximize vaccine efficacy. TGF-β1 production by MDSCs not only directly suppresses other immune cells (e.g., CD8^+^ T cells) but also feeds into an autocrine loop to enhance development of additional tumor-associated MDSCs (86). MDSCs have also been shown to induce expansion of immunosuppressive, tumor-specific Tregs (87). Consequently, whether or not the MDSC activation is mediated by IL-13 from type II NKT cells, neutralizing TGF-β could interrupt autocrine and paracrine TGF-β loops driving suppression of tumor immunity (86, 88). Simultaneously, a type I NKT cell agonist could aid in the maturation of DCs, the suppression of MDSCs, and the enhancement of NK and CD8^+^ T cells to restore the anti-tumor immune response, essentially killing several birds with one stone.

There are additional mechanisms of NKT cell-mediated tumor suppression as well. In a K7M2 mouse osteosarcoma model, it was found that 88% of NKT cell-deficient mice allowed for the rejection of osteosarcoma primary tumors, compared to a 24% rejection rate in WT mice containing both type I and type II NKT cells (89). Tumor rejection was shown to be CD8^+^ T cell-dependent, and CD1d KO mice that rejected the tumor had significantly higher numbers of tumor infiltrating lymphocytes. While previously TGF-β and IL-13 mediated such suppression, TGF-β and IL-13 were not the drivers of immunosuppression in this model, and even though the exact mechanism was not revealed, this study highlighted that alternative pathways exist for CD1d-dependent NKT cell-mediated immunosuppression. For instance, we recently found that sulfatide-reactive type II NKT cells inhibit proliferation of naïve, CD4^+^ T cells (90), whereas they may not suppress memory CD4^+^ T cells (43). Because CD4^+^ T cells aid CD8^+^ T cells, it is possible that in situations where NKT cells interrupt CD8^+^ T cell-immunity and tumor rejection, NKT cell suppression of naïve CD4^+^ T cells may contribute (90).

Although in the majority of tumor settings the immunosuppressive role of NKT cells has been ascribed to type II NKT cells, while type I are associated with an improved anti-tumor immune response, there are exceptions (91–93). Paradoxically, activated type I NKT cells have been shown to support immunosuppressive Tregs through IL-2 production, though the NKT cells are subsequently suppressed by Tregs in a cell-contact-dependent manner (94). Two studies of note have reported that type I NKT cells directly suppress tumor immunity in hematological malignancies. In a model of Burkett’s-like B cell lymphoma, Jα18 KO mice deficient in type I NKT cells had significantly fewer splenic tumors than WT or CD1d KO mice. While stimulation of type I NKT cells with α-GalCer did not increase tumor burden, it decreased tumor-specific CD8^+^ T cells (91). Given a T cell lymphoma cell line transfected with CD1d, NKT deficient mice had augmented CTL activity and greater survival rates than WT mice (92). Accordingly, the authors suggested that tumor expression of CD1d might shift the NKT cell response toward a Th2 skewed anti-inflammatory reaction, which produces more IL-13, TGF-β, and inhibition of CTL and NK cell activity.

### Cross regulation

Type I and type II NKT subsets have been shown to not only differentially impact cell populations but also cross-regulate one another. This immunoregulatory axis was realized when Jα18 KO mice, which lacked type I NKT cells showed higher susceptibility to tumor growth than WT mice in a CT26 tumor pulmonary metastasis model, whereas CD1d KO mice deficient in both types showed strong resistance against the tumor. This led us to believe that type I NKT cells may be acting to reduce the suppressive effect of type II NKT cells (27). Sulfatide activation of type II NKT cells *in vivo* enhanced tumorigenesis, and abolished or reduced the beneficial clinical effects of α-GalCer when the two were administered together. Thus, it appeared that type II NKT cells may also suppress type I NKT cell activation (26, 27). The latter effect likely results from decreased pro-inflammatory cytokine secretion by type I cells. *In vitro*, α-GalCer-induced IFN-γ, IL-2, and IL-4 production was inhibited upon addition of the type II antigen sulfatide (27, 95). Whereas this effect may not always be entirely due to type II mediated suppression, but occur partially or in some cases because sulfatide competes with α-GalCer in binding CD1d (95), this competitive binding mechanism was excluded in the settings described by Ambrosino et al. (27) In a model of concanavalin A-induced hepatitis, it was demonstrated that activation of sulfatide-reactive type II NKT cells and plasmacytoid DCs in the liver contributed to the anergy, or hyporesponsiveness of type I NKT cells (96). It is unclear if this mechanism is the same in the tumor setting. Nonetheless, the discovery that protective type I and suppressive type II NKT cells cross-regulate one another defines an NKT regulatory axis, provides a better understanding of the tumor immunity equilibrium, and opens a window of opportunity to exploit that knowledge in a clinical setting.

## Clinical Applications

In addition to the positive results of harnessing NKT cell potential in pre-clinical studies, clinical observations suggested diminished NKT cell function in cancer. For example, reports have found patients battling multiple myeloma and solid tumors to have significantly fewer NKT cells than healthy donors (74, 97). Several studies showed that the number of type I NKT cells in tumors or in peripheral blood correlated with lower rates of lymph node metastases, highly significant rates of overall and disease-free survival and served as an independent predictor for patient prognosis (98–100). Taken together with pre-clinical studies in mice, data imply that stimulation of type I NKT cells might be a useful strategy for cancer therapy in human beings. However, no clinical response was observed in the original clinical trial using α-GalCer/KRN7000 to treat solid tumors (97). The less-than-expected efficacy in human trials was believed to have multiple contributing factors. Potential problems include insufficient drug delivery, too low NKT cell numbers at the time of treatment, induction of NKT cell anergy, existence of natural anti-α-gal-antibodies, or other confounding factors like cross regulation of beneficial NKT cell activity (Table 1).

**Table 1:** **Clinical dilemmas, possible mechanisms, and potential solutions**.

Clinical dilemma	Potential mechanism	Potential solution
Low NKT cell number	Innate predisposition	NKT cell adoptive transfer therapy
	Result of tumor	iPS NKT cell adoptive transfer therapy
Suboptimal response to type I NKT cell antigen	Inadequate drug delivery or route	α-GalCer-pulsed APCs
		Tumor cells loaded with α-GalCer (tumor/gal)
		Nanovectors with α-GalCer
	Combination of Th1 and Th2 cytokine release	Antigen enhancement (e.g.,α-C-GalCer, NU-α-GalCer)
		Clarification of which NKT cell subset is being stimulated (i.e., NKT1, NKT2, NKT17, etc.)
α-GalCer-induced anergy	PD-1/PD-L1 expression	PD-1/PD-L1 blockade
	Cbl-b expression	Cbl-b silencing
	Innate quality of α-GalCer antigen	Use of alternative antigen e.g., β-ManCer
	Unknown	α-GalCer-pulsed APCs
		Intradermal administration of NKT cell agonist
		Nanoparticle delivery
Anti-α-Gal antibody neutralization of α-linked NKT cell antigens	Natural anti-α-Gal-antibodies	Antigen without α-Gal sugar moiety, e.g., β-ManCer

### Adequate stimulation

Because pre-clinical data showed APCs preloaded with antigen better augmented NKT cell-based anti-tumor responses than free α-GalCer, α-GalCer-pulsed APCs were administered intravenously to patients (101), reviewed in Ref. (102). These α-GalCer-pulsed APCs are delivered to the lung parenchyma, where they activate type I NKT cells and initiate both direct and indirect anti-tumor effects. Multiple phase I and II clinical studies using monotherapy of α-GalCer-pulsed APCs on non-small cell lung cancer (NSCLC) and head and neck squamous cell carcinoma (HNSCC) reported objective anti-tumor responses, albeit at low frequency. The combination of this therapy with infusion of pre-activated type I NKT cells in HNSCC resulted in objective responses in about 40% of patients, and a majority of those treated displayed enhanced numbers of IFN-γ-expressing type I NKT cells in peripheral blood (103). Alternative routes of administration for α-GalCer-pulsed APCs are under investigation (104, 105), and it will be interesting to see whether these NKT cell-specific immune responses can be optimized for other cancer types as well.

The immense variability in NKT cell number from patient to patient may also contribute to variability in clinical response. One potential solution to overcome this issue is NKT cell adoptive transfer therapy. A recent study by Fujii et al. (106) establishing induced pluripotent stem (iPS) cells that can generate unlimited numbers of NKT cells with adjuvant activity may realize this type of therapy.

### Anergy

Anergy may also hinder the beneficial and/or lasting effects of NKT cell-mediated treatment (107). NKT cell anergy refers to a tolerance mechanism following repeated antigen stimulation in which the lymphocyte remains in a hyporesponsive state for an extended period of time (108). New protocols, which prevent anergy induction are being employed, such as intradermal administration or nanoparticle delivery of the NKT cell agonist (105, 109). The exact mechanism of NKT cell long-term functional anergy induction is under investigation by numerous groups (107, 110–114). Marked increase in programed death-1 marker (PD-1) expression after α-GalCer stimulation prompted multiple studies on the role of PD-1 in type I NKT cell anergy, as PD-1 is an exhaustion marker and negative regulator of T cell antigen receptor signaling. However, whether PD-1 blockade can prevent or reverse anergy remains controversial (110, 111, 115). Also, it was demonstrated that the PD-1/PD-L1 pathway was essential for CD4^+^ type II NKT cells to suppress CD4^+^ T cells (116). With the observations that PD-1 is upregulated by the exhausted CD8^+^ T cells (117), and has been shown to be a mechanism for tumor evasion (118, 119), targeting PD-1 is an attractive therapeutic approach. PD-1/PDL1 blockade may not only prevent or reverse type I NKT cell anergy and decrease suppressive activity of type II NKT cells on CD4^+^ T cells but it may also augment tumor immunity by preserving CD8^+^ T cells. Combining anti-PD-1 treatment with an NKT cell agonist may also be beneficial, for that could stimulate NKT cell-mediated DC maturation, NK cell activity, and cross-priming of those “rescued” CD8^+^ T cells. This is particularly interesting when considering that ongoing clinical trials using anti-PD-1 in treatment for melanoma, NSCLC, and renal cell carcinoma show early evidence for some efficacy, but could be improved (120).

Additionally, PD-1 and the E3 ubiquitin ligase, casitas B-lineage lymphoma-b (Cbl-b) are both regulated by Egr2/3, which is overexpressed during α-GalCer-induced anergy (74). Cbl-b is increased when type I NKT cells become anergic and Cbl-b deficiency permits rescue of IFN-γ but not IL-4 production in anergic NKT cells (112). Therefore, blocking Cbl-b with small molecule or peptide antagonists may prevent anergy induction by α-GalCer.

### Natural anti-α-gal-antibodies

Another potential roadblock to α-GalCer treatment involves the fact that human and non-human primates cannot make α-linked sugar moieties and have natural anti-α-gal-antibodies (121). These antibodies may neutralize the α-linked component of the drug. Although it was originally believed that an alpha-linked sugar moiety was necessary for glycosphingolipids to activate type I NKT cells, a recent study showed that a ceramide with a beta-linked sugar moiety could induce a comparable protective effect (67). The β-glycolipid analog β-mannosylceramide (β-ManCer) acts through an independent mechanism from that of the α-linked KRN7000 (nitric oxide synthase/TNF-α pathways, as opposed to primarily IFN-γ). It not only protected mice against tumors, but also synergized with α-GalCer, so lower doses of both drugs could be used (67). Additionally, β-ManCer failed to induce long-term NKT cell anergy (122). In contrast to α-GalCer, β-ManCer may achieve protection with less cytokine production, decreasing toxicity, less likelihood of anergy, and without potential neutralization by anti-α-gal-antibodies. Thus, this new class of type I NKT cell agonists represented by β-ManCer may overcome several of the problems that could be interfering with clinical efficacy of α-GalCer and its close analogs.

### DCs and vaccines

A recurring theme seems to suggest that a single agent α-GalCer may not be sufficient for cancer treatment. However, the NKT cell-mediated activation of NK, B, CD4^+^, and CD8^+^ T cells suggests many opportunities to use NKT cell agonists as vaccine adjuvants. As mentioned, type I NKT cells enable DCs to cross-prime CD8^+^ T cells. Coadministration of α-GalCer with peptide or protein antigens enhances the priming and boosting of CD8^+^ T cells (10–12). A recent approach with vaccines involving nanovectors, CD1d-containing endosomes that serve as nanoparticle carriers of α-GalCer, is an attractive method to stimulate NKT cells. The carriers are taken up by APCs, and because they release α-GalCer more slowly than current methods (soluble infusion or DC-pulsing); they require lower drug amounts for similar biological effects (123). The microspheres can be decorated with specific antibodies to focus them on particular targets, and particles can be engineered to enhance cross-priming of CD8^+^ T cells by targeting CD8^+^DCs (123).

A separate approach to enhance cross-priming of CD8^+^ T cells that differs from simply administering antigen alongside an NKT cell agonist has been tried in cancer. In a pre-clinical study, tumor cells were loaded with α-GalCer (named tumor/gal), irradiated, and infused intravenously (124). Tumor/gal cells were lysed by NKT and NK cells in an innate fashion but NKT cells also reacted to the α-GalCer/CD1d complex on splenic DCs, which had taken up debris from lysed tumors. This allowed DCs to mature and tumor antigen was cross-presented to CD8^+^ T cells to induce a tumor-specific CTL response, which protected mice from tumor challenge. More research is needed to determine which types of cancer cells to inject for tumor/Gal therapy. For instance, because cancer stem cells or cancer-initiating cells may drive malignancy and immune-escape, injecting glycolipid-coated, irradiated cancer stem cells could enhance the therapeutic effect (124).

### Chemotherapy

Combining an NKT cell agonist with certain types of chemotherapy is another option for harnessing the potential of NKT cell-mediated protection (59, 125). In multiple myeloma, a small cohort of patients had an enhanced immune response when α-GalCer-stimulated NKT cells were administered in conjunction with lenolidomide (an approved treatment for multiple myeloma) (125). The therapy caused an activation of type I NKT cells and consequent induction of activated NK cells and eosinophils.

## Conclusion

The role that NKT cells play in tumor immunity is becoming increasingly clear, especially as we develop a more accurate and holistic understanding of how NKT cell subsets differentially impact the innate and adaptive immune cell populations. Initial attempts to harness the immune enhancing effects of type I NKT cells in the context of cancer were met with limited success during translation into the clinic. However, that clinical research has encouraged further investigation and broader understanding of NKT cell subsets in cancer. The immunological potential of type I NKT cells *in vivo* is being optimized through antigen enhancement, tactical delivery, and even *ex vivo* expansion. Insight on the suppressive mechanisms of type II NKT cells has informed methods of intervention to restore tumor immunity (e.g., anti-TGF-β). Finally, taking advantage of the adjuvant effect mediated by NKT cells in vaccines and chemotherapy treatment has proven to augment tumor immunity in animal models. While there is optimism that NKT cells could be an effective tool in enhancing the anti-tumor immune response, an incomplete understanding of NKT cell subsets has hampered the progress of translating the basic and pre-clinical NKT cell studies into the clinic. The successful strides noted in this review will be enhanced further with a greater understanding of what drives context-specific interactions between NKT cells and other cell populations, as well as by further clarification of the role of differentiated subsets within type I (NKT1, NKT2, NKT17, NKTreg, NKT_FH_), as well as the heterogeneous subset of non-invariant or type II NKT cells.

## Conflict of Interest Statement

The authors declare that the research was conducted in the absence of any commercial or financial relationships that could be construed as a potential conflict of interest.
